# An Equality-Based Approach to Analysing the Global Food System’s Fair Share, Overshoot, and Responsibility for Exceeding the Climate Change Planetary Boundary

**DOI:** 10.3390/foods11213459

**Published:** 2022-11-01

**Authors:** Yan Li, Ajishnu Roy, Xuhui Dong

**Affiliations:** 1 School of Geographical Sciences and Remote Sensing, Guangzhou University, Guangzhou 510006, China; 2 Centre for Climate and Environmental Changes, Guangzhou University, Guangzhou 510006, China

**Keywords:** planetary boundaries, food system, GHG emissions, fair share, carbon neutrality, global

## Abstract

The climate catastrophe is being caused by human effects on earth system processes that are surpassing several planetary boundaries. This crisis is driven significantly by the global food system. It has been increasing over recent years, yet food systems are essential in upholding food and nutrition security. This study proposed a novel method for enumerating national contributions to the cessation of the climate crisis by approximating nations’ aggregate greenhouse gas (GHG) emissions from food systems, within the equitable and sustainable planetary boundaries of climate change. This study included 221 nations, which were grouped as per their human development index (HDI) categories, income groups, and continental locations. During 1990–2018, the annual fair share, overshoot of emissions, and collective historical responsibility in the world of each country were assessed. There was a 22.52% increase in overshooting of GHG emissions from the global food system, starting in 1990. A group of 15 countries, including Brazil, China, Indonesia, and the U.S.A., were responsible for >67% of global overshoot. The primary liability is borne by countries with upper-, middle-, and high-income economies, and high to very-high HDI groups, as well as Asia and South America. Countries such as India, China, the Democratic Republic of the Congo, and others have steadily increased their share of responsibility over the last 28 years. More than 76% of countries in the world, mostly from Africa, Europe, and Asia, proved to be absolute overshooters. After contextualising the study’s findings, the global food system’s decarbonization and its limits were discussed; some recommendations for prospective research were also offered. It appears that academics, governments, and policymakers should start concentrating more on reshaping and redesigning the global food system to be climate-friendly (i.e., a carbon-neutral food system), whilst being able to fairly allocate food and nutrition security to achieve long-term Sustainable Development Goal 2 (SDG 2).

## 1. Introduction

Food systems (FS) are comprised of all stakeholders and their interconnected value-adding activities associating with the production, aggregation, manufacturing, dissemination, consumption, and disposal of foodstuffs [1]. These emanate from cultivation, forestry, or fishing, as well as components of the wider economic, societal, and environmental systems in which they have been engrained. Food systems consist of sub-systems (such as cultivation, processing, packaging, etc.) and intersect with other critical components (such as energy, trade, health, wellbeing, etc.) (see Glossary). A sustainable food system (SFS) ensures food and nutritional security for everyone, as well as safeguarding the economic, societal, and environmental foundations essential to the provision of food and nutrition security to subsequent generations [2] (see Glossary). Therefore, food systems should be beneficial in the long haul (economic sustainability), have substantial societal benefits (social sustainability), and have a neutral or positive effect on the surrounding resources and ecosystem (environmental sustainability) [3]. At least eight of the seventeen UN SDGs are strongly connected to food systems [4].

Food systems account for 19–29% of global anthropogenic GHG emissions, generating 9800–16,900 Mt CO_2_-e in 2008 [5]. Agricultural production accounts for 80–86% of overall food system emissions, when considering indirect emissions related to land-cover change. Food contributes to 26% (13.7 billion tonnes of CO_2_-e) of global GHG emissions, and the consequences of the lowest-impact animal products often outweigh those of vegetable equivalents, indicating the need for dietary reform [6]. The majority of GHG emission from are from livestock and fisheries (31%), followed by crop production (27%), land use (24%), and supply chain (18%). In 2010–2050, as a result of a population explosion and a change in income levels, in the absence of technological advances and focused mitigation efforts, the global food system’s environmental consequences might expand by 50–90% [7]. Current trends in global food systems would impede the 1.5 °C objectives from being met, and by the end of the century, the target of 2 °C would be endangered [8]. In that case, a substantial decrease (>60%) is needed in 2020–2100 from business-as-usual emissions. Emissions from the global food system were 12–19 Gt CO_2_-e y^−1^ for the period 2008–2017 [9,10]. A shift towards more sustainable production and consumption patterns might provide 10.2 billion (instead of only 3.4 billion) people within four planetary boundaries (PBs) (viz. land-system change, biosphere integrity, nitrogen flows, and freshwater use) [11] (see Glossary). For China’s food production to remain within national and provincial PBs in 2030, a 47–99% decrease (from 2011) in phosphorus, nitrogen, and GHG emissions, blue-water consumption, and land usage is required [12].

The global food system is essential for the well-being of all humans, yet remains reasonably poorly comprehended. Owing to an exploding population, a transition from a traditional to a sustainable food system is the need of the hour. Among others, food systems are highly impacted by climate change. The prevailing paradigm of safe operating space (SOS) yields a clear macro-understanding regarding environmental sustainability (see Glossary). Hence, the interlinkage of SDG 13 (climate change) with the global food system has been explored. The philosophical objective is to turn the present food system’s rising GHG emissions into GHG removals, thus embracing carbon neutrality in the global food system (see Glossary). Due to the quantity of GHG emissions released within the farm and on agricultural land, agriculture is a substantial contributor to climate change from food systems. However, because different post-farm activities, such as those throughout the supply chain, retail, consumption, and waste disposal, among others, add up, the influence of the overall food system on climate change is considerably larger. Hence, there is also a need to point out targets for either decarbonization or decoupling emissions from food systems of the highest contributing countries.

This study aims to (1) formulate a methodology to assess the role of nations in exceeding the safe operating space of climate change regarding food systems; then (2) allocate historical responsibilities to four income groups, four HDI categories (see Glossary), and six continents; and (3) identify priority sectoral emissions of nations with higher responsibility, which we hope would be helpful to future researchers in decarbonizing the global food system. This work (1) resides in the interacting zone of the global food system and climate change (SDG 13), (2) delves deeper into connecting the planetary boundary framework (viz., the climate change boundary) with the global food system, and (3) explores the inclusive equity-based allocation of the historical responsibility of nations in the world.

## 2. Methodology

### 2.1. Quantifying the Safe Climate Space of the Global Food System

The framework of planetary boundaries (PB) was first published in 2009 [13,14]. Then, a substantial update was composed [15]. There have been some noteworthy papers on PBs related to agriculture and/or food systems, especially concerning climate change boundaries. The global carbon budget (see Glossary) value is 4.7 Gt CO_2_-e y^−1^ (range: 4.3–5.3 Gt CO_2_-e y^−1^) for non-CO_2_ emissions [16]. Another work [17] has shown that the contribution of agriculture to total anthropogenic GHG emissions is nearly 11% (5.0–5.8 Gt CO_2_-e y^−1^), which rises to up to 25% when agriculturally-driven land-cover change is considered. For agricultural production, the boundary was introduced as 0.65 Wm^−2^ [18]. Others [19,20] have set the climate change PB of the global food system as 5 Gt CO_2_-e y^−1^ (range: 4·7–5·4 Gt CO_2_-e y^−1^) in 2050, which was also supported by another work [21]. Another study [12] set 5.4 Gt CO_2_-e y^−1^ (range: 4.9–6.1 Gt CO_2_-e y^−1^) as a safe climate space (SCS, i.e., safe operating space related to climate change) (see Glossary). The lower limit (4.7 Gt CO_2_-e y^−1^) of the SCS of the global food system was conservatively chosen in this study.

### 2.2. Quantifying Fair Share, Overshoot, and Responsibility

Some metrics for excess GHG emissions were assessed concerning *equity-based fair sharing* to finalise the amount to which a country’s resource consumption or emissions contribute to global climate breakdown. The focus was on some metrics of excess GHG emissions, assessed concerning *equity-based fair sharing*. The World Bank provided the population data [22]. The global food system’s GHG emissions dataset from the EDGAR-Food database (2022) was used, based on two recent assessments [23,24]. The global food system’s SCS was distributed based on each country’s population as a share of the global population; in other words, on an equal fair-share basis, in keeping with the principle of climate commons, amalgamating recently proposed methodologies [25,26,27]. This has been used to evaluate overall carbon emissions [25,26] and material footprint [27] (see Glossary), but has not been repurposed for anything else; more precisely, it has not been used for carbon emissions from the global food system, which matters significantly [28]. Using this method, each country’s fair share (see Glossary) of the annual climate change PB, vis-à-vis the global food system over 28 years (1990–2018), was calculated. The unit of responsibility here is the nation, concerning time, not individual citizens’ responsibility. A fair share of GHG emissions from the global food system for any country, or country group or region, was obtained by multiplying the SCS of the global food system with the share of that unit’s population in the world. These equitable distributions do not remain constant over time, as populations shift annually within both countries and the world.

From that, the overshoot of GHG emissions regarding the global food system was computed as the excess GHG emissions from the global food system over its designated fair share (Equation (1)).

Food system’s GHG overshoot = actual food system GHG emissions from the area − fair share of food system GHG emissions of that area(1)

The undershooting nations, i.e., those whose total food GHG emissions are less than their fair share of the climate change limit, were inferred. They carry no responsibility for climate change; rather, they hold a climate credit against overshooting countries, while overshooting countries owe them a climate debt (see Glossary). This method allowed for the calculation of liability for climate change, which served as a guide for assigning accountability for the resulting losses. The proportion of each country’s overshoot to the overall global overshoot was used to determine responsibility. Undershoot was set to zero in all years, ensuring that undershoot in one year does not compensate for overshoot in another year(s). The cumulative overshoot of each nation was estimated by adding national overshoots from 1990 to 2018 (Equation (2)).

Annual overshoot _(1990)_ + ⋯ + Annual overshoot _(2018)_ = Cumulative overshoot of food system’s GHG emissions _(1990–2018)_ (2)

Finally, the national culpability for overall excess GHG emissions from the global food system was quantified by dividing each nation’s cumulative food GHG overshoots by the sum of all nations’ cumulative food GHG overshoots (i.e., cumulative global overshoot of carbon budget).

## 3. Results

### 3.1. Temporal Change of GHG Emissions and Overshoots Globally from Food Systems (1990–2018)

In 28 years (1990–2018), 459.14 Gt of CO_2_-e GHG emissions were generated globally from food systems. Annually, this changed from 14.68 to 16.92 Gt (i.e., a 15.31% increase). CO_2_ was the major contributor to food system emissions (56.14%) in 2018, just as it was in 1990. CH_4_ and N_2_O, on the other hand, contributed 23.04% and 15.36%, respectively. On average, there was an annual increase of 80.28 Mt CO_2_-e GHG emissions. Based on the chosen safe climate space (SCS) of the global food system (viz. 4.7 Gt CO_2_-e y^−1^), there was an overshoot of 9.98 Gt CO_2_-e GHG emission in the very first year of estimation (1990). By 2018, this increased to 12.22 Gt CO_2_-e. This means there was a 22.52% increase in overshoot of GHG emissions from the global food system. There was not a single year when global GHG emissions from food systems undershot the SCS. On the contrary, when all the annual overshoots on a global scale, over 28 years, are added up, it becomes 322.84 Gt.

### 3.2. Overshoots of GHG Emissions and Annual Comparative Share of Exceeding SCS at the Country Level

**Figure 1:**
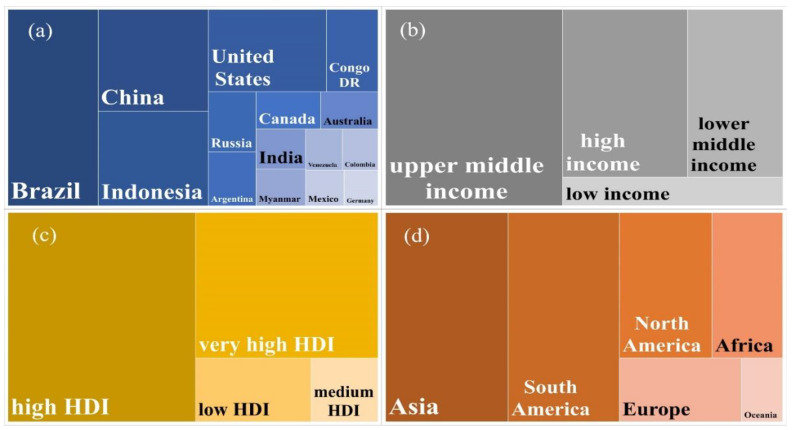
Allocating distribution of cumulative overshoot of GHG emissions from food systems (1990–2018). GHG emissions from (**a**) 15 countries, (**b**) four income groups (as per World Bank), (**c**) four HDI categories (as per HDR, 2020), and (**d**) six continents.

On a sub-global scale, Brazil (52.85 Gt), China (33.36 Gt), Indonesia (30.95 Gt), the U.S.A. (29.03 Gt), Congo D.R. (12.62 Gt), Russia (8.58 Gt), Argentina (7.65 Gt), Canada (7.18 Gt), Australia (6.36 Gt), and India (5.89 Gt) are the top ten countries with the highest collective overshoot (1990–2018) (Table 1, Figure 1a). This means there is a mixture of countries from the global north and south.

**Figure 2:**
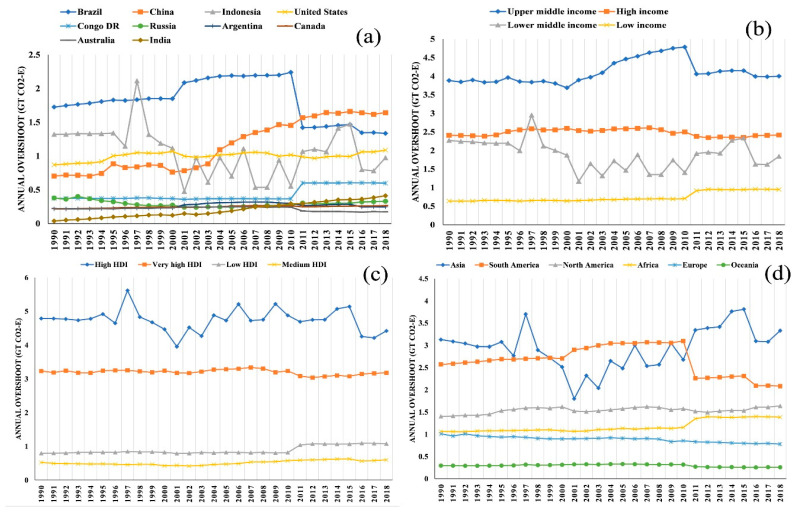
Annual overshoot of national or regional fair share of GHG emissions from food systems (1990–2018). Annual overshoots from (**a**) 10 countries, (**b**) four income groups (as per World Bank), (**c**) four HDI categories (as per HDR, 2020), and (**d**) six continents.

When considering the annual overshoot of GHG emissions from the global food system at the country level (Figure 2a), the top ten countries with the highest share of annual overshoot (in 1990) were Brazil (1.72 Gt), Indonesia (1.32 Gt), the U.S.A. (0.87 Gt), China (0.7 Gt), Russia (0.379 Gt), Congo D.R. (0.375 Gt), Australia (0.22 Gt), Canada (0.219 Gt), Argentina (0.218 Gt), and India (0.03 Gt). Over 28 years (from 1990 to 2018), four of these countries showed a decrease in their annual overshoot, which are Indonesia (26.1%), Australia (21.66%), Brazil (22.56%), and Russia (12.9%). The remaining six, India (968.13%), China (133.72%), Congo D.R. (59.51%), the U.S.A. (24.97%), Canada (21.3%), and Argentina (13.17%), showed an increase in their annual overshoot. Among these top ten countries, there were some interesting trends, easily recognisable in Figure 2. Brazil showed a rise in 2001 and then a fall in 2011. China showed a small spike in 1994, and then again in 2003. Since then, China has steadily increased the amount of its annual overshoot. Indonesia showed a significant increase in 1997 and maintained its ups and downs during the whole study period. The U.S.A. showed a slow increase, with three distinct minor spikes in 1995, 2000, and 2016. Congo D.R. (Kinshasa) maintained a steady level of annual overshoot for 28 years, with a single major uprise in 2011. India showed a very slow yet gradual rise right from the start (1990).

**Figure 3:**
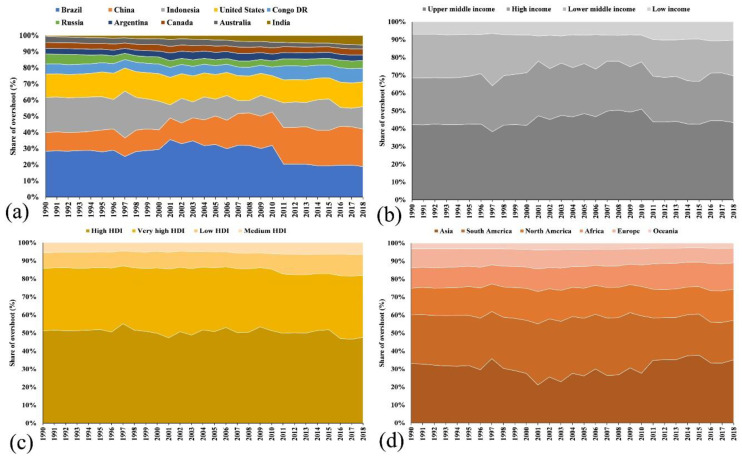
Annual share of overshoot (%) of excess GHG emissions from the global food system (1990–2018). The annual shares of (**a**) 10 countries, (**b**) four income groups (as per World Bank), (**c**) four HDI categories (as per HDR, 2020), and (**d**) six continents.

Next, considering the annual comparative share (%) of exceeding the SCS of the global food system, the proportion of total anthropogenic GHG emissions attributed to food systems varied greatly among nations and regions. At the country level (Figure 3a), the top ten countries with the highest share (1990) in global overshoot were Brazil (17.28%), Indonesia (13.24%), the U.S.A. (8.72%), China (7.04%), Russia (3.8%), Congo D.R. (3.76%), Australia (2.23%), Canada (2.19%), Argentina (2.18%), and India (0.38%). Over 28 years, four of them showed an increase in their contribution to global shares, namely India (771.78%), China (90.75%), Congo D.R. (30.18%), and the U.S.A. (1.99%). The remaining six showed a decrease in comparison to their 1990 level, namely Indonesia (39.69%), Brazil (36.8%), Australia (36.06%), Russia (28.91%), Argentina (7.62%), and Canada (0.99%).

### 3.3. Overshoots of GHG Emissions from the Food System and Annual Comparative Share of Exceeding SCS at Continent Scale (1990–2018)

On the continental scale (Figure 2d), there is an equal number of continents that increased and decreased in 2018, in comparison to 1990. Africa (30.14%), North America (16.79%), and Asia (6.43%) showed an increase in their annual overshoot, whilst Europe (22.8%), South America (18.93%), and Oceania (12.62%) showed a decrease. Among these continents, Europe and Oceania showed a slow yet gradual shrinkage. Contrary to North America, which remained reasonably stable, South America showed an increasing phase (1990–2010) with one surge (2001) followed by two dips, a major one in 2011, and another minor one in 2016. Asia showed the highest fluctuation, with two major surges (1997 and 2015) and a few dips (1996, 2001, 2003, 2005, 2007, and 2016).

On the continental scale (Figure 3d), the largest share of global overshoot (1990) was from Asia (31.35%), followed by South America (25.79%), North America (14.08%), Africa (10.68%), Europe (10.14%), and Oceania (2.98%). Over the following 28 years, except for Africa (6.21% increase), all continents decreased, namely Europe (36.99%), South America (33.84%), Oceania (28.69%), Asia (13.13%), and North America (4.67%), compared to 1990 levels.

### 3.4. Overshoots of GHG Emissions and Annual Comparative Share of Exceeding SCS at Group Levels

At the income group level (Figure 1b), the aggregate highest overshoots were from upper-middle income (119.04 Gt), followed by high (71.89 Gt), lower-middle (54.85 Gt), and low income (21.59 Gt). At the level of HDI groups (Figure 1c), the highest overshoots were from high HDI (137.46 Gt), followed by very-high (92.84 Gt), low (25.73 Gt), and medium HDI (14.93 Gt). On the continental scale (Figure 1d), the highest overshoots were from Asia (85.27 Gt), followed by South America (77.31 Gt), North America (44.79 Gt), Africa (34.14 Gt), Europe (25.86 Gt), and Oceania (8.71 Gt). The continent that has the highest responsibility for exceeding the SCS of the food system is Asia (26.41%), followed by South America (23.94%), North America (13.87%), Africa (10.59%), Europe (8.01%), and Oceania (2.69%).

At the income group level (Figure 2b), it can be seen that the highest degree of annual overshoot was seen in the upper-middle group (3.88 Gt), followed by high (2.41 Gt), lower-middle (2.27 Gt) and low-income (0.63 Gt) in 1990. Over 28 years, except for lower-middle-income (18.84% decrease), all income groups showed an increase, namely low (48.95%), upper-middle (3.01%), and high income (0.16%). Among these income groups, high- and low-income groups’ annual overshoot remained almost unchanged except for a spike in low-income in 2011. Lower-middle countries showed an upsurge in 1997 and fluctuated since then. The upper-middle group showed a rise in 2000, followed by an increasing phase, then a dip in 2010, and then remained nearly steady.

At the level of HDI groups (Figure 2c), the highest degree of annual overshoot (1990) was seen in the high HDI group (4.78 Gt), followed by very-high (3.23 Gt), low (0.79 Gt), and medium HDI (0.52 Gt). Over the 28 years, two groups showed a decrease, namely high (7.68%) and very-high HDI (1.6%), whilst the remaining two groups showed an increase in annual overshoot, namely low (35.85%) and medium HDI (14.41%). Among the HDI groups, the very-high and medium HDI groups remained relatively stable since 1990. The low HDI group showed a minor spike in 2011 and remained uplifted since. Only the high HDI group showed a sharp spike in 1997 and fluctuated since.

At the income group level (Figure 3b), the highest share of global overshoot (1990) belonged to the upper-middle income group (38.9%), followed by high income (24.16%), lower-middle income (22.75%), and low income (6.39%). Over time, except for low income (21.57% increase), all groups decreased, namely lower-middle income (33.76%), high income (18.24%), and upper-middle income (15.92%).

At the level of HDI groups (Figure 3c), the largest share of global overshoot (1990) belonged to the high HDI group (47.96%), followed by very-high (32.4%), low (7.97%), and medium (5.25%) HDI. Over the following years, except for the low HDI group (10.88% increase), all groups decreased, namely the high (24.65%), very-high (19.69%), and medium HDI (6.62%) groups, in comparison to 1990 levels.

### 3.5. Absolute Overshooters at Different Scales and Levels

*Absolute overshooters* are those countries that have overshot their corresponding fair share from the first year, i.e., there is no year with an undershoot (see Glossary). Based on absolute overshooting, 76.01% of countries (i.e., *n* = 168) belong to this category. Based on continental distribution, most are from Africa (25.59%), Europe (24.4%), and Asia (22.61%). The remainder are from North America (12.5%), South America (7.73%), and Oceania (7.14%). Based on the geographical location of countries from diverse continents, the majority of countries from all continents are in the absolute overshooting category. These are from South America (almost 100%), North America (91.3%), Oceania (85.71%), Europe (80.39%), Africa (79.62%), and Asia (77.55%). Based on HDI categories, most of these countries belong to very-high (36.77%) and high (27.74%) HDI groups. The residuals are from the medium (18.06%) and low (14.41%) HDI categories. Based on income categories, most of these countries belong to the high (34.37%) and lower-middle (26.25%) groups. The rest is from the upper-middle (25.62%) and low (13.75%) income groups. Including these 168 absolute overshooting countries, there are 206 countries (i.e., 93.21%) that have shown GHG overshooting characteristics for at least a year. This indicates that there are only 15 countries in this world which are undershooting, i.e., don’t bear any responsibility for exceeding food system SCS in 28 years (1990–2018).

**Figure 4:**
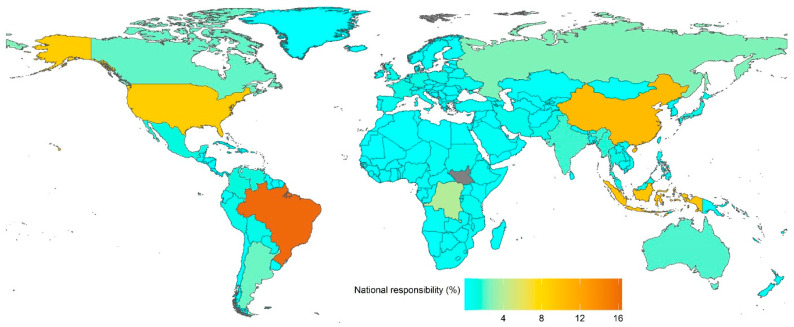
National level of responsibility over 28 years (1990–2018) for overshooting fair share of safe climate space with food systems.

When the accountability for exceeding the food system’s SCS is taken under consideration, the countries with the highest obligations are Brazil (16.37%), China (10.33%), Indonesia (9.58%), the U.S.A. (8.99%), Congo D.R. (3.9%), Russia (2.65%), Argentina (2.37%), Canada (2.22%), Australia (1.97%), and India (1.82%) (Figure 4). BRICS countries are answerable for 31.71% of the cumulative overshoot. EU-27 countries are accountable for a 7.5% overshoot. G-8 countries are liable for 18.37% of overshoot.

### 3.6. Who Bears Responsibility for Emissions from Global Food Systems?

Now the results generated from this study need to be contextualized. Countries of mixed features have historically contributed drastically towards food systems’ GHG exceeding SCS. For example, when the top 15 countries are considered (1990–2018), they have traditionally been responsible for more than 67% of the total. Of these countries, most are from very-high or high HDI categories, i.e., representatives from medium and low HDI countries are surprisingly low. Similarly, when income is considered (according to the World Bank), it is seen that the majority are from the upper-middle- and high-income groups, but not from lower-middle- or low-income economies. Considering their geographical locations, it is clear that most are from Asia and South America. Again, the contribution from continents such as Europe, North America, Oceania, etc. is remarkably lower. On one hand, countries from higher-income or HDI, or richer continents, are not exclusively responsible for exceeding the safe climate space of food system GHG. On the other hand, countries with just the opposite features (from lower-income or HDI or poorer continents) are also not singularly answerable.

### 3.7. GHG Emissions of Food System from Different Substances and Stages

**Figure 5:**
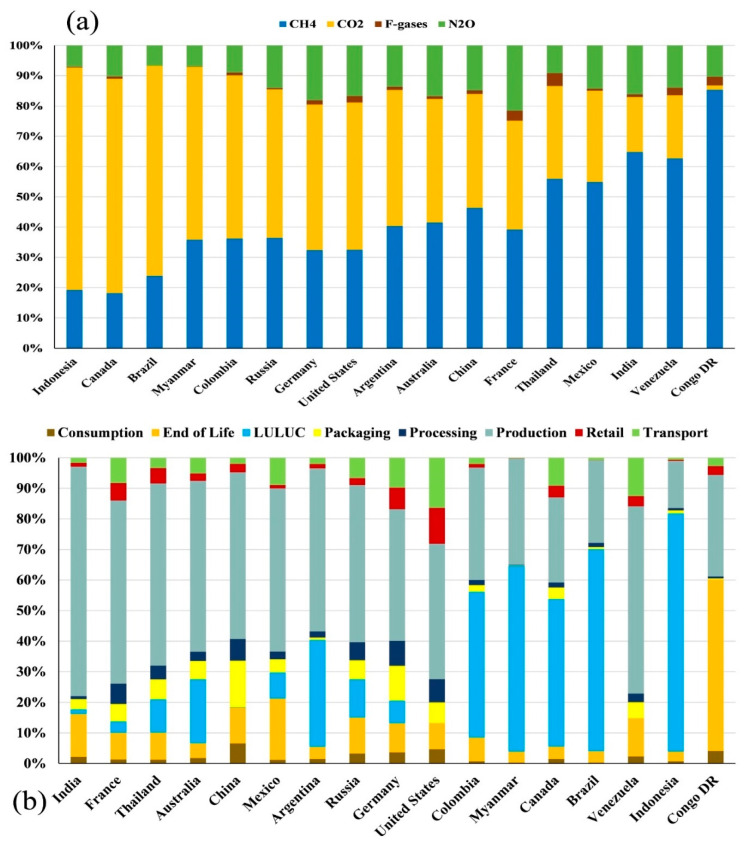
Distribution of GHG emissions for the 17 nations of highest historical responsibility (>1%). It shows the share (%) of total historical emissions. (**a**) GHG emissions from four substances—CH_4_, CO_2_, F-gases, and N_2_O. (**b**) GHG emissions from eight sectors—Consumption, End of Life, LULUC (Land Use and Land-Use Changes), Packaging, Processing, Production, Retail, and Transport.

When the GHG emissions from different substances are considered (Figure 5a), the share of CH_4_ (%) ranges from <20% (19.26%, Indonesia; 18.15%, Canada; 3.22%, Congo D.R.) to 54–65% (64.82%, India; 55.94%, Thailand; 54.92%, Mexico). For CO_2_ (%), this ranges from <20% (18.11%, India; 7.86%, Venezuela; 0.056%, Congo D.R.) to 69–73% (69.32%, Brazil; 70.83%, Canada; 73.44%, Indonesia). When the share of N_2_O (%) is considered, this ranges from <7% (6.98%, Myanmar; 6.77%, Brazil; 5.25%, Venezuela; 0.39%, Congo D.R.) to 18–21% (18.13%, Germany; 21.56%, France). For F-gases, this ranges from <0.25% (Indonesia, Congo D.R., and Myanmar) to 3–4% (Thailand, France, and the USA).

When the GHG emissions from different stages of the global food system are considered (Figure 5b), the contribution of production (%) ranges from 1–15% (Congo D.R. and Indonesia) to 59–75% (Thailand, France, and India). The share of land use and land-use changes, LULUC (%), accounts for between <3% (China, India, and France) and >60% (Myanmar, Brazil, and Indonesia). The other six sectors have a relatively low contribution. This indicates a very heterogeneous picture of the global food system’s emissions across countries. From these calculations, an outline of the most significant substances and sectors of GHG emissions of the top 17 contributing nations in the world can be drawn. They are Brazil (CO_2_ and LULUC), China (CH_4_ and production), Indonesia (CO_2_ and LULUC), the U.S.A. (CO_2_ and production), Congo D.R. (CH_4_ and end of life (based on available data where LULUC is absent)), Russia (CO_2_ and production), Argentina (CO_2_ and production), Canada (CO_2_ and LULUC), Australia (CH_4_ and production), India (CH_4_ and production), Myanmar (CO_2_ and LULUC), Venezuela (CH_4_ and production (LULUC emission data is absent)), Colombia (CO_2_ and LULUC), Mexico (CH_4_ and production), Germany (CO_2_ and production), Thailand (CH_4_ and production), and France (CH_4_ and production).

## 4. Discussion

We hypothesised that a group of countries which are reliant on agriculture to feed their higher populations, or to generate income via heavy levels of trade (either export or import) of food (or agricultural) products, possibly bear higher liability. In particular, countries that produce or trade carbon-heavy agricultural commodities or engage in other high carbon-emitting food system activities (excluding farm production) could shoulder this responsibility. A few recently published works support this hypothesis. According to estimates of trade-adjusted agricultural emissions of food products [29,30,31], emissions are mostly influenced by a nation’s consumption behaviour and agricultural emission intensities, in comparison to those of its trading partners. The production-based and trade-adjusted emissions accounting approaches differ significantly in absolute terms, particularly for the largest agricultural exporters and importers, as well as areas where significant amounts of emission-intensive products, such as ruminant meat, milk products, and rice, are present. This work demonstrates that not only agricultural production on the farm, but also the final fate of food products (after the trade), explains a high degree of the responsibility of countries found in this study. As per another very recent study [32], the total food-miles equate to around 3.0 Gt CO_2_-e, showing that transportation contributes to about 19% of all food-system emissions (stemming from transport, production, and land-use change). Nearly twice as much GHG is generated during their production in the form of global trade routes, which accounts for 36% of food-mile emissions. This means that the globalised supply chain of the global food system bears significant accountability for the rising involvement of these countries. In a broader scope, various socioeconomic, as well as behavioural, factors drive these countries to reach a state where they are at the forefront of surpassing the safe climate space of the global food system, for many years. Another study [24] reached a somewhat similar conclusion. There should be two major ways left to solve this: either decoupling population growth and food-related emissions or decarbonizing the global food system. Since not all aspects of food-system emissions are human-controlled, and keeping current socio-economic development patterns in mind, decoupling might prove to be rather difficult. Hence, a greater focus should be given to the decarbonization of the global food system. Now, decarbonization needs to attain specificity in target, meaning which country, sector (of the food system), and substance (gas)-related emissions to focus on. From this analysis, 17 nations have been identified as having the highest level of national responsibility (1.06–16.37%) for historically exceeding the SCS of the food system.

In recent years, there have been a handful of papers that have focused on decarbonization overall, as well as specifically on the decarbonization of the food system. A recent study [25] established that the global north and Annex I (i.e., significantly industrialised) nations are responsible for 92% and 90% of total national overshoots, respectively, in terms of historical cumulative overall emissions. The European Union (EU-28) and the G-8 countries are responsible for 29% and 85%, respectively. Since this study focuses on total emissions, meaning not specific to any sector (such as food systems), the results differ from our study. Another relevant study [27] concluded that high-income countries are responsible for 74% of the global overshoot of material use, while China and the rest of the global south are responsible for only 23%. These results also differ as they focus on material use. However, these studies clearly establish a notion that countries holding comparatively smaller populations and greater economic power in the world bear the highest responsibility for overall climate change or excess material usage. However, in the present study, the nations with higher responsibility hail from the upper-middle and high-income nations in Asia and South America. Since agriculture is a large part of the food system, agricultural economies are bound to be higher emitters, or, gradually, overshooters. In this way, if they are the end consumers, they should be affluent, by now, when food security is taken into consideration. However, it can be observed that a only small portion of them fall into the global average, and the majority fall even lower in global food security [33], or are not free from hunger [34]. These distinct results of the present study also justify the need to delve deeper into the system or sector level, along with overall analyses.

In terms of CO_2_ reduction from food systems, energy consumption on farms is a significant source of emissions [35]. Another group [36] opined that disruption, innovation, nonlinear change in human behaviour, etc., are very often left out of model-based decarbonization assessments and scenarios. A socio-technical approach was suggested [37] to address the complexity of the deep decarbonization problem and demonstrate how co-evolutionary interactions between technological and socio-cultural entities might hasten low-carbon transitions in a multitude of sectors. The authors proposed a carbon-law framework for decarbonization (halving gross anthropogenic CO_2_ emissions per decade). There have recently been a few papers and discussions on the degrowth perspective for an emission-neutral food system [38,39]. Reducing and redistributing income has a limited chance of mitigating GHG emissions from agriculture and land-use change. At the border between managed and unmanaged land, improved agricultural land management, reduced deforestation, and natural ecosystem conservation will need to be accelerated [40]. One study [41] estimated the GHG emissions of the U.S.A. food system (3800 kg CO_2_-e/capita in 2010), where cities directly contribute nearly 66%. A worldwide network should be built for modelling, managing, and mapping agriculture, biodiversity, trade, nutrition, etc. [42]. The authors proposed three pillars to focus on: efficient and resilient agricultural systems; biodiversity preservation and restoration; and food security and nutritious diets. After compiling a thorough scoping evaluation of 701 papers related to decarbonizing the food and beverage industry, 78 promising transformational technologies were identified [43]. Another study [38] demonstrated that a systemic, quantifiable food system transition, which is composed of a complementary coupled strategy of the equitable income distribution (degrowth) and efficient resource allocation, can achieve a steady-state food-system economy that is net GHG-neutral by 2100, while improving nutritional outcomes. Another study [39] argued that degrowth may integrate decreased animal protein consumption, carbon pricing, and income redistribution to promote climate mitigation in the food system. A just low-carbon transition for food systems has been proposed [44], and the criteria for it consist of six dimensions: distributive justice (right to essential goods, labour justice, livelihood opportunities, etc.); cosmopolitan justice (global fairness; intergenerational justice); justice for the environment and non-human beings (ecological integrity; justice for animals); procedural justice (just processes); recognition justice (respectful treatment of others); and capacities (non-discriminatory capacity development). Based on the evaluation of >60 known low-emission and carbon-sequestration scenarios, strengthening food systems might reduce their emissions from 21.4 to −2.0 Gt CO_2_-e y^−1^ and fulfil rising food demand without relying on carbon offsets [45]. There have been some recent studies on the drivers of GHG emissions from food systems [46,47,48,49,50,51,52] and possible ways to mitigate them [53,54,55].

Combining previous research findings with the findings of this work, it can be understood that the food industry will require specific sectoral energy-efficiency and decarbonization measures to mitigate climate change. That means countries that shoulder comparatively higher liability for food GHG emissions should be chosen, their explicit sector identified, and then targeted, tailored decarbonization actions should be studied that operate simultaneously on two agendas: cutting down emissions to reach a safe operating level of the global food system, now as well as in the near future (i.e., emission efficiency within SOS), whilst also achieving equitable allocation of food and nutrition (i.e., inclusive sufficiency food security).

The present study has five inadequacies that merit discussion. *One* is that the starting year of the analysis had a significant impact on snowballing climate-change boundary-overshoot accounting. The year 1990 was chosen since the EDGAR-Food database also began in 1990. The picture of the world’s food system and trade during the colonial era may have been utterly different as a result of land enclosures, deforestation, pollution, forced food appropriation, and other factors. Beginning in 1990 essentially nullifies whatever extra GHG emissions that the world’s colonializing countries may have accumulated before that time. Countries of the Global South, such as Brazil, China, Indonesia, Congo D.R., Argentina, India, etc., on the other hand, are penalised for the same activity because it occurred within the analysis period and during a time of overall GHG emissions. The impact of the richer, better-HDI, higher-income, and colonising nations of the global north would have been a few times more than those countries in the contradictory situation, if the research periods had gone as far back as the 1700s or 1800s. It can be suggested that, if possible, the EDGAR-Food database would reach back to at least the 1950s, the accepted onset of the Anthropocene.

A *second* shortcoming relates to the aggregated nature of the climate change boundary, particularly given how complicated the global food system is—there are at least 14 stages [23]. The analysis may be strengthened by breaking down the boundary into safe operating limits for each of these stages and, as a result, assigning responsibility for more precise environmental constraints resulting from the unsustainable nature of the global food system. However, academic research has just progressed to the point that an SOS for climate change can be offered for the whole food system. We anticipate that more studies in this area in the future will be able to estimate the safe operating limits of climate change for the global food system at various stages.

The possible *third* downside is the annualised nature of the food system’s climate change border. No matter how much time is spent in violation of the climate change border, it remains exceeded. If too much GHG is emitted from the global food system in the past, less scope of emission (i.e., the carbon budget of the food system) will be available in future generations if we don’t opt for destabilising the earth system. Instead of a cumulative period budget (as in [25]), this annualised carbon budget of the food system aids in obtaining insight into both a horizontal view (what happened everywhere in a specific year) and a longitudinal view (summary of what has happened everywhere over the study period).

A *fourth* possible hitch relates to the absence of a land-system change boundary in this study. Food is chiefly sourced from agriculture, and hence, agriculture-driven land-use change and the specific SOS concerned should have been associated with this study. This study was focused on the carbon budget of the food system, i.e., carbon was the currency here instead of land. To estimate land-system change from the food system would have required another dimension of work, fairly distinct from this work (though converging on the identical theme of the global food system), which would have resulted in a complex fusion of two separate perspectives in a single work, which might have yielded difficulty in targeted communication of the outcomes of this study. We suggest formulating such a database, which can explore the global food system and land nexus and derive insights from it.

A *fifth* snag relates to deciphering the specific drivers that caused a spike or a dip in food GHG in countries. This problem would necessitate a different type of study, possibly through the political economy of the food system. However, that is outside the scope of this study. Nonetheless, it can be advocated that taking into account each of these sudden (either sharp spike or dip), as well as gradual, changes in food GHG emissions of countries, at least for those that are major contributors, and organising tailored studies for each country in the future, because one universal study that fits all and explains the situation of all the dissimilar countries is not possible, would be beneficial.

## 5. Conclusions & Policy Recommendations

In accordance with the paradigm of PBs and fair allocation of food, the equitable fair-shares methodology used in the study delivers a dependable way for assessing national to regional scale accountability for climate change from food system GHG emissions. The findings offer direction for choosing fair methods of establishing liability for losses resulting from the food system’s role in climate change. These findings highlight what can be called a process of *food system colonization*. Only a handful of nations have historically contributed much more than their fair share of GHG emissions to the global exceedance of the planetary threshold for climate change caused by the food system.

Keeping in mind the looming state of global food security, decarbonization of the global food system is required fast and urgently. If not, on one hand, the food system would not be able to sufficiently feed the world; on the other hand, the food system could feed the world at the cost of exceeding planetary safe operating limits, which would then take a toll on the complex situation of the stability of the earth system in this Anthropocene. Another thing is that, if we don’t offer significant weightage to stakeholders, i.e., socio-economic and cultural context, whatever method we come up with for decarbonizing the global food system couldn’t be either implemented or maintained, and hence, the decarbonization would flop. Having said that, several significant projects are currently underway, relating to the environmental effects of the food system, as well as the socioeconomic security of food and nutrition. National emissions from food systems have been globally networked through routes of globalisation and the supply chains of food systems. Though all anthropogenic GHG emissions from food systems put weight on the climate, a certain amount of essential GHG emissions is inevitable to maintain human society’s functioning, and nations with larger populations will require higher baseline GHG emissions than those with lower populations. To improve quantification of food-related emissions in national reporting and identify mitigation options across the whole food system, with the help of the EDGAR-Food database, a comparative outline is presented for effective approximation of countries that are overshooting SOS of climate change, as well as those that bear historical responsibility. However, since the global food system is networked via the global food trade, many countries should not be identified, marked and left on their own. Several things need further investigation. So far, what has been achieved from previous and current studies is at the identification phase, on a global to national scale. Researchers now need to probe deeper into operational scales. *First*, by delving into the sub-national scale, i.e., assessing GHG emissions from a state or province to the district, city/town to village level. *Second*, by employing direct filtration of carbon-intensive food systems in any place. Then again, sectoral quantification of emissions is needed to churn out specific leverage points of a food system that is responsible for higher emissions. Then, embedded in their socioeconomic situation, ways to decarbonize those focal points should be found without creating any adverse impact on the place or the people involved, i.e., stakeholders of that food system. We are hopeful that this work will enhance a much-required understanding of the global food system, which would guide future research toward achieving carbon neutrality in the global food system.

There are a few suggestions that could be provided based on outcomes of this study. The top overshooting countries, namely Brazil, China, Indonesia, the U.S.A., Congo D.R., Russia, and Argentina, should immediately focus on reducing emissions from food systems. To do that, they need to start keeping track of sub-national and trade-based emissions from food systems, so that more focus can be given to underlying levels of organisations. A food watch-like committee should be stared, which would keep the academic and policy related actions and their outcomes organized. It needs to have a special focus on nations of upper-middle and high income, high and very-high HDI, Asia, and south America, as these are the groups that shoulder the highest responsibility. More research is needed into place-based and trade-based emissions from food systems, their causes, and ways to minimize them, both at intra- and international levels. The food system-related polices of these overshooting countries need to be aligned into scientifically informed (i.e., evidence-based) policies, possibly in the form of a dashboard or toolbox for food system emissions.

## Figures and Tables

**Table 1:** List of top overshooting countries (*n* = 15) for climate change fair shares of the food system.

Rank	Country (Overshooters or Climate Debtors)	Cumulative Emission (Gt CO_2_-e)	Cumulative Overshoot (Gt CO_2_-e)	Overshoot (%)	Mean Annual Overshoot (Gt CO_2_-e)	Responsibility in the World (%)
1	Brazil	56.71	52.85	1369.17	1.88	16.37
2	China	60.72	33.36	121.92	1.19	10.33
3	Indonesia	35.69	30.95	652.95	1.1	9.58
4	The U.S.A.	35.22	29.03	468.98	1.03	8.99
5	Congo D.R. (Kinshasa)	13.78	12.62	1087.93	0.45	3.9
6	Russia	11.69	8.58	275.88	0.3	2.65
7	Argentina	8.47	7.65	932.92	0.27	2.37
8	Canada	7.86	7.18	1055.88	0.25	2.22
9	Australia	6.8	6.36	1445.45	0.22	1.97
10	India	29.61	5.89	24.83	0.21	1.82
11	Myanmar	6.46	5.44	533.33	0.19	1.68
12	Venezuela	4.99	4.45	824.07	0.159	1.38
13	Colombia	5.25	4.37	496.59	0.156	1.35
14	Mexico	6.32	4.1	184.68	0.14	1.27
15	Germany	5.36	3.61	206.28	0.12	1.11

## Data Availability

The authors confirm that the data, used in this study, is available in the public database: EDGAR-Food (https://edgar.jrc.ec.europa.eu/edgar_food, accessed on 5 August 2022).

## Glossary

**Term**	**Explanation**
Absolute overshooters	The nations that have overshot their own fair shares of emissions beginning from the first year of calculated period.
Carbon budget	The total cumulative net worldwide anthropogenic CO2 emissions that, after accounting for the impact of other human climatic forcings, would result in limiting global warming to a particular level with a given likelihood.
Carbon-neutral (or carbon neutrality)	The state of balance between emission (from carbon source) and accumulation of carbon (from carbon sinks) in any form, i.e., perfect equilibrium of input and output in terms of amount of carbon.
Climate debt	The amount that one country is alleged to owe another country for the harm that its disproportionately significant climate change contributions have created.
Fair share	The understanding that all peoples have equal rights to resource consumption or emission within the limits of the planet’s carrying capacity.
Food system (FS)	The interrelated systems and processes that have an impact on agriculture, food, nutrition, health, and community development. Growing, harvesting, processing, packaging, shipping, selling, consuming, distributing, and disposing of food and food-related things are all included in this.
Human development index (HDI)	A statistical composite index that ranks nations into four stages of human development (viz., very-high, high, medium, and low) based on life expectancy, education (mean and expected years of schooling), and per capita income indicators.
Material footprint (MF)	The relationship between global material extraction and a nation’s domestic end demand. It is the total of the material footprints for metal ores, non-metal ores, metal ores, biomass, and fossil fuels.
Planetary boundaries (PB) concept	Ten environmental boundaries that will allow mankind to progress and flourish for many more centuries.
Safe climate space (SCS)	The safe operating space related to the planetary boundary of climate change.
Safe operating space (SOS)	A scenario in which Earth’s ability to maintain human life is not in jeopardy and human cultures’ capacity for adaptation is not necessarily overburdened.
Sustainable food system (SFS)	A food system that ensures everyone has access to food security and nutrient-dense food while maintaining the economic, social, and environmental foundations necessary to provide food security and nutrition for both current and future generations.
